# Histopathological Diagnosis of Opisthorchiasis in an Immigrant

**DOI:** 10.4269/ajtmh.2010.10-0218

**Published:** 2010-10-05

**Authors:** Dennis Tappe, Ralph Triefenbach

**Affiliations:** Institute of Hygiene and Microbiology, Würzburg, Germany; Institute for Pathology and Cytology, Limburg, Germany

A 49-year-old woman from Kazakhstan, who had immigrated to Germany ~5 years before, developed acute abdominal pain of the right upper quadrant. Cholecystolithiasis was suspected clinically. White blood count showed 7,130 leukocytes/µL with 10% eosinophils. C-reactive protein level was elevated (19 mg/dL), and liver function tests showed a raised gamma-GT of 59 U/L. Cholecystectomy was performed, however, no gallstones were found. Histopathological examination of the removed gallbladder revealed a slight chronic inflammation of the lamina propria. In a Luschka's duct, helminth parasites consistent with hepatic trematodes of the genus *Opisthorchis* were found (Figures 1 and 2). The eggs seen in the trematode's uterus were much smaller than those of the cosmopolitan liver fluke *Fasciola hepatica*. Stool examination for helminth ova was not performed. Treatment with praziquantel (75 mg/kg/d in 3 doses) was advised. Opisthorchiasis is caused by the small liver flukes *Opisthorchis felineus* in the Russian Federation, Ukraine, and Kazakhstan, and *Opisthorchis viverrini* in Thailand, Lao, Cambodia, and Vietnam.^2^ Approximately 1.2 million people are infected by *O. felineus*, the most likely parasite in the case described here, and 12.5 million people are at risk.^3^ The infection is acquired by eating raw or undercooked cyprinoid fish, which contains metacercariae of the trematodes.^2^ Not only humans, but also cats, dogs, and possibly many fish-eating mammals, act as definitive hosts. Adult flukes reside in small- and medium-sized intrahepatic bile ducts, and occasionally also in the gallbladder, extrahepatic bile ducts, and in the pancreatic duct.^2^ In contrast to infection with *O. viverrini*, a group 1 carcinogen,^4^ many patients infected by *O. felineus* suffer from fever and hepatitis-like symptoms in the early stage of infection,^5^ and no association with cholangiocarcinoma formation has been established. In chronic infections, patients may present with cholangitis and liver abscess because of biliary obstruction.^2^ Diagnosis is achieved by detection of eggs in feces, however, species-specific diagnosis on the basis of egg morphology is difficult.^5^ Polymerase chain reaction detecting DNA of the adult parasite in stool may be helpful^2^ and sometimes the parasites are detected histopathologically *in situ*, as in the case described here.

**Figure 1.**
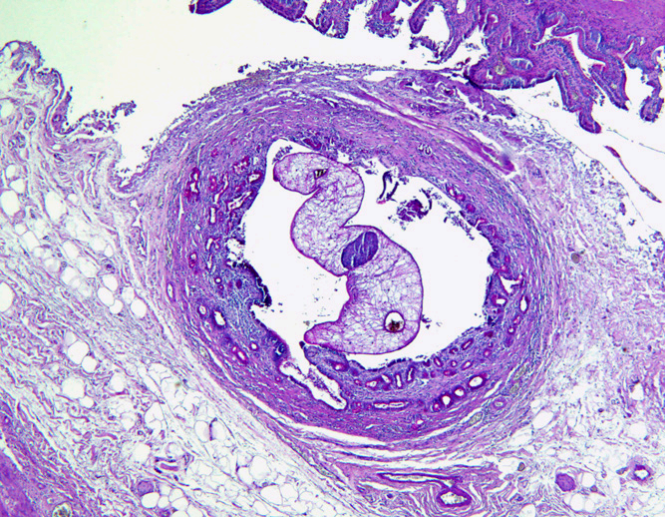
*Opisthorchis* sp. in Luschka's duct from an immigrant from Kazakhstan. Cross section through posterior third of the parasite. In the center of the trematode the ovary is visible, whereas laterally the paired ceca can be seen. Note absence of vitellaria (compared with Figure 2) in this section plane, which favors the diagnosis of *Opisthorchis felineus* over the very similar *Opisthorchis viverrini*.^1^ Periodic acid-Schiff stain, original magnification ×40. This figure appears in color at www.ajtmh.org.

**Figure 2.**
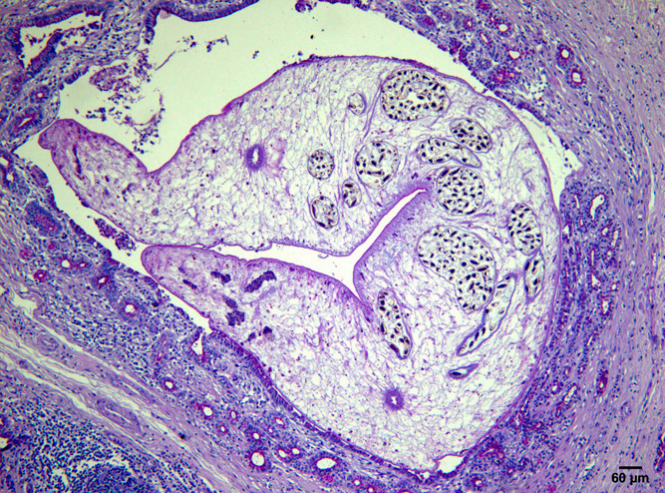
Close-up of *Opisthorchis* sp. Multiple branches of the egg-filled uterus are visible, flanked by the paired ceca on this cross section through the midbody of the aspinous fluke. Eggs measure 32 × 14 µm on average (length: width ratio of 2.28) and are thus consistent with *Opisthorchis felineus*.^1^ Sparse vitellaria are found on the lateral extremes of the parasite on this section. Slight chronic inflammation of the hosts's lamina propria without marked eosinophilic infiltrate can be seen. Periodic acid-Schiff stain, original magnification ×100. This figure appears in color at www.ajtmh.org.

## References

[1] Mas-Coma S Bargues Castello MD Marty AM Neafie RC 2000 Hepatic trematodiases Meyers WM Neafie RC Marty AM Wear DJ Pathology of Infectious Diseases, Volume 1, Helminthiases Washington, DC Armed Forces Institute of Pathology, American Registry of Pathology 69 92

[2] Marcos LA Terashima A Gotuzzo E 2008 Update on hepatobiliary flukes: fascioliasis, opisthorchiasis and clonorchiasis Curr Opin Infect Dis 21 523 530 18725803 10.1097/QCO.0b013e32830f9818

[3] Keiser J Utzinger J 2005 Emerging foodborne trematodiasis Emerg Infect Dis 11 1507 1514 16318688 10.3201/eid1110.050614 PMC3366753

[4] Bouvard V Baan R Straif K Grosse Y Secretan B El Ghissassi F Benbrahim-Tallaa L Guha N Freeman C Galichet L Cogliano V WHO International Agency for Research on Cancer Monograph Working Group 2009 A review of human carcinogens–Part B: biological agents Lancet Oncol 10 321 322 19350698 10.1016/s1470-2045(09)70096-8

[5] Keiser J Utzinger J 2009 Food-borne trematodiases Clin Microbiol Rev 22 466 483 19597009 10.1128/CMR.00012-09 PMC2708390

